# CAAStools: a toolbox to identify and test Convergent Amino Acid Substitutions

**DOI:** 10.1093/bioinformatics/btad623

**Published:** 2023-10-16

**Authors:** Fabio Barteri, Alejandro Valenzuela, Xavier Farré, David de Juan, Gerard Muntané, Borja Esteve-Altava, Arcadi Navarro

**Affiliations:** IBE, Institute of Evolutionary Biology (UPF-CSIC), Department of Medicine and Life Sciences, Universitat Pompeu Fabra. C. Doctor Aiguader 88, Barcelona 08003, Spain; BarcelonaBeta Brain Research Center, Pasqual Maragall Foundation, C/ Wellington 30, Barcelona 08006, Spain; IBE, Institute of Evolutionary Biology (UPF-CSIC), Department of Medicine and Life Sciences, Universitat Pompeu Fabra. C. Doctor Aiguader 88, Barcelona 08003, Spain; BarcelonaBeta Brain Research Center, Pasqual Maragall Foundation, C/ Wellington 30, Barcelona 08006, Spain; Genomes for Life-GCAT Lab, GermanTrias i Pujol Research Institute (IGTP), Camí de les Escoles, s/n, Badalona 08916, Spain; IBE, Institute of Evolutionary Biology (UPF-CSIC), Department of Medicine and Life Sciences, Universitat Pompeu Fabra. C. Doctor Aiguader 88, Barcelona 08003, Spain; IBE, Institute of Evolutionary Biology (UPF-CSIC), Department of Medicine and Life Sciences, Universitat Pompeu Fabra. C. Doctor Aiguader 88, Barcelona 08003, Spain; Institut d’Investigació Sanitària Pere Virgili (IISPV), Hospital Universitari Institut Pere Mata, Universitat Rovira i Virgili. Avda. Josep Laporte, 2 – Planta 0 – E2 color taronja, Reus 43204, Spain; Centro de Investigación Biomédica en Red en Salud Mental (CIBERSAM), Av. Monforte de Lemos, 3-5. Pabellón 11. Planta 0. Madrid 28029, Spain; European Molecular Biology Laboratory, Meyerhofstraße 1, Heidelberg 69117, Germany; IBE, Institute of Evolutionary Biology (UPF-CSIC), Department of Medicine and Life Sciences, Universitat Pompeu Fabra. C. Doctor Aiguader 88, Barcelona 08003, Spain; BarcelonaBeta Brain Research Center, Pasqual Maragall Foundation, C/ Wellington 30, Barcelona 08006, Spain; Institució Catalana de Recerca i Estudis Avançats (ICREA) and Universitat Pompeu Fabra, Pg. Lluís Companys 23, Barcelona 08010, Spain; Center for Genomic Regulation (CRG), The Barcelona Institute of Science and Technology, C. Doctor Aiguader N88, Barcelona 08003, Spain

## Abstract

**Motivation:**

Coincidence of Convergent Amino Acid Substitutions (CAAS) with phenotypic convergences allow pinpointing genes and even individual mutations that are likely to be associated with trait variation within their phylogenetic context. Such findings can provide useful insights into the genetic architecture of complex phenotypes.

**Results:**

Here we introduce CAAStools, a set of bioinformatics tools to identify and validate CAAS in orthologous protein alignments for predefined groups of species representing the phenotypic values targeted by the user.

**Availability and implementation:**

CAAStools source code is available at http://github.com/linudz/caastools, along with documentation and examples.

## 1 Introduction

Convergent Amino Acid Substitutions (CAAS) provide important insights into the genetic changes underlying phenotypic variation (Zhang and Kumar 1997, Rey *et al.* 2019). Recent examples include the identification of genes potentially involved in marine adaptation in mammals (Foote *et al.* 2015) and the convergent evolution of mitochondrial genes in deep-sea fish species (Shen *et al.* 2019). Notably, in 2018, Muntané *et al.* identified a set of 25 genes involved in longevity in primates (Muntané *et al.* 2018). A few years later, a similar analysis for a wider phylogeny retrieved 996 genes associated with lifespan determination in mammals (Farré *et al.* 2021). While these analyses often need to be tailored for each particular phenotype and phylogeny, all CAAS detection and validation strategies reported in the literature share some common steps (Rey *et al.* 2019). First, researchers select the species to compare for CAAS analysis and split them into two or more groups according to the phenotype of interest. The criteria to select these groups can be quite diverse: for instance, groups can be formed by species having diverging values of a given continuous trait, or by species sharing different adaptations, like terrestrial and marine mammals (Foote *et al.* 2015). The second step consists in linking amino acid substitutions with each group. Here, different approaches can be used, such as identifying identical substitutions for the same amino acid (Besnard *et al.* 2009, Chabrol *et al.* 2018), detecting topological incongruencies (Li *et al.* 2008), variations in amino acid profiles (Rodrigue *et al.* 2010, Rey *et al.* 2018), or relying on consistent patterns of groups of amino acids in different groups of species (Zhang *et al.* 2014, Muntané *et al.* 2018, Farré *et al.* 2021). The third step consists in testing the significance of the results. Molecular convergence is a noisy process because spurious CAAS may occur at random in the absence of relationships with phenotypes or selective forces (Xu *et al.* 2017). To overcome this, researchers have adopted different strategies, mostly based on the idea that adaptive CAAS tend to exceed convergent noise. The delta Site-Specific log-Likelihood Score (ΔSSLS), for instance, is a method that consists in comparing the CAAS likelihood for different phylogenetic topologies (Castoe *et al.* 2009, Parker *et al.* 2013, Wang *et al.* 2013). Another approach uses bootstrap resampling tests to evaluate whether the number of detected CAAS is larger than expected by chance (Muntané *et al.* 2018, Farré *et al.* 2021). Alternatively, some authors have adopted a strategy that consists in quantifying the convergent noise and focus on the detection of Convergence on Conservative Sites (Xu *et al.* 2017, He *et al.* 2020). In spite of all these contributions, there is still no consensus approach. Some authors question whether phenotypic convergence matches genome-wide molecular convergence (Zou and Zhang 2015b), or whether adaptive substitutions outnumber random CAAS (Thomas and Hahn, 2015; Zou and Zhang 2015a). Access to free software tools that are specifically designed to retrieve CAAS will allow the wider research community to compare and validate different strategies, boosting future methodological developments in the field of phylogenetic analysis.

Here we present CAAStools, a toolbox to identify and validate CAAS in a phylogenetic context. CAAStools is based on the strategy applied in our previous studies (Muntané *et al.* 2018, Farré *et al.* 2021) and implements different testing strategies through bootstrap analysis. CAAStools is designed to be included in parallel workflows and is optimized to allow scalability at proteome level.

## 2 Implementation

**Figure 1.**
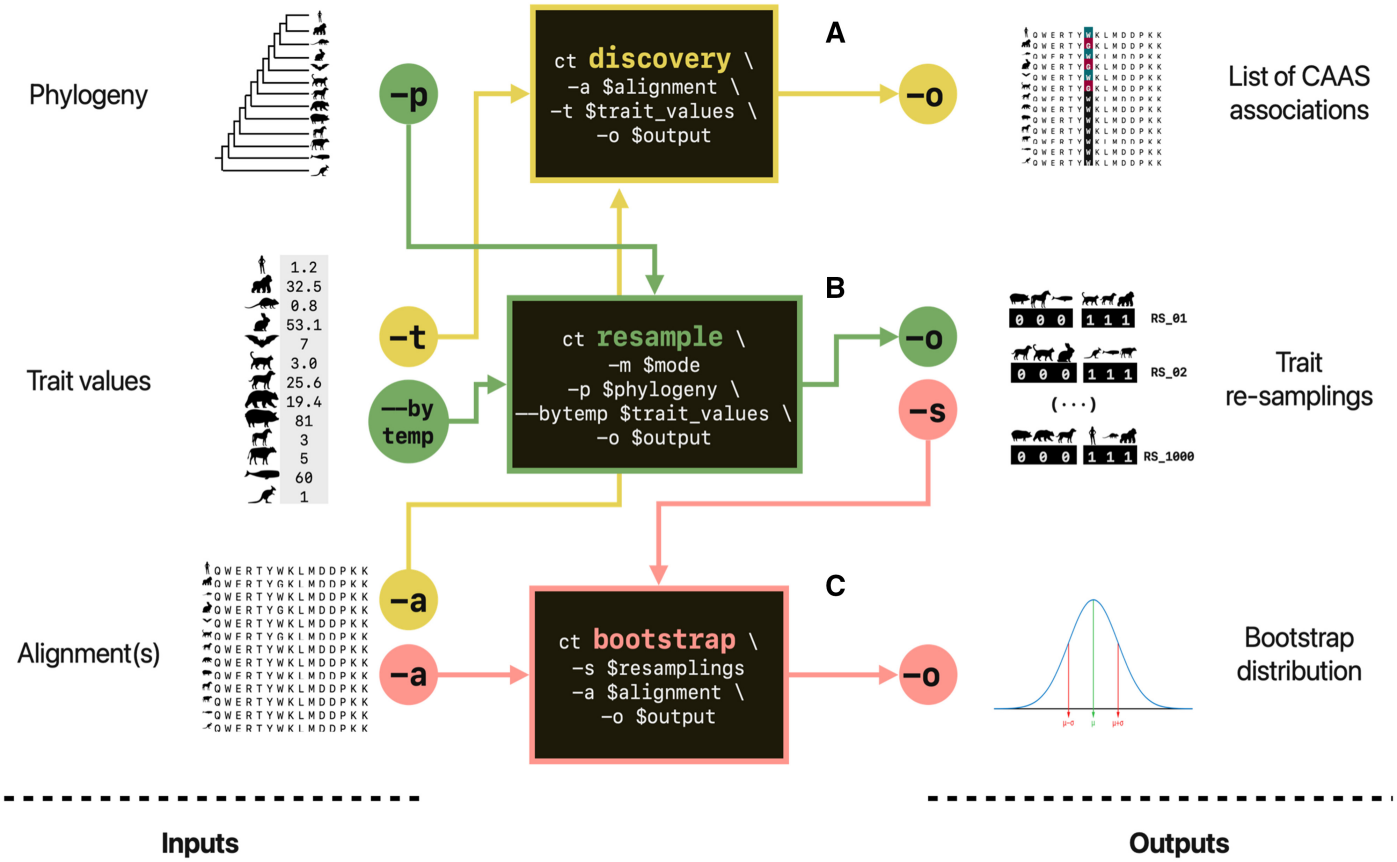
CAAStools layout. The three tools of the CAAStools suite rely on three pieces of information; a phylogenetic tree, the trait information, and an amino acid MSA. The discovery tool (A) detects the CAAS between two groups of species that are defined by the user on the basis of trait values. The resample tool (B) performs *n* trait resamplings in different modalities, on the bases of the phylogeny and the trait value distributions. The output of this resampling is processed by the bootstrap tool (C) that elaborates a bootstrap distribution from the MSA. All the tools can be executed independently.

CAAStools is a multi-modular python application organized into three tools. The outline of the suite is presented in Fig. 1. The discovery tool is based on the protocol described in Muntané *et al.* (2018) and Farré *et al.* (2021). This approach identifies CAAS between two groups of species in an amino-acid Multiple Sequence Alignment (MSA) of orthologous proteins. These groups are named Foreground Group (FG) and Background Group (BG). Collectively, the two groups are called Discovery Groups (DG), as they represent the base for CAAS discovery. The CAAS identification algorithm scans each MSA and returns those positions that meet the following conditions: First, the FG and the BG species must share no amino acids in that position. Second, all the species in at least one of the two discovery groups (FG or BG) must share the same amino acid. The combination of these two conditions determines a set of different mutation patterns that the tool identifies as CAAS. Details on these patterns are provided in Supplementary Table S1.

Finally, CAAStools calculates the probability of obtaining a CAAS in a given position compared to randomized DGs, corresponding to the empirical *P*-value of the predicted CAAS in that position. This *P*-value represents a quantification of the convergent noise (Shahoua *et al.* 2017) that is associated with a specific position. The details of this calculation are presented in Supplementary Section S3. The Resample tool sorts species into *n* virtual DGs (resamplings) for bootstrap analysis according to different combination strategies. This tool enables bootstrap analyses based on CAAS excess or likelihood (Castoe *et al.* 2009, Muntané *et al.* 2018, Farré *et al.* 2021). In a *Naive* modality, the probability of every species being included in a DG is considered identical and independent. This feature allows for bootstrap analyses aimed at quantifying convergent noise. However, species are phylogenetically related, biasing their probability of sharing a phenotype or amino acid. To address these phylogenetic dependencies CAAStools includes two other testing strategies. In the *Phylogeny-restricted* modality, the randomization can be restricted to some taxonomic orders or defined clades. These clades will match the ones of the species included in the DGs. In the *Brownian motion* modality, resampling is based on Brownian Motion simulations. The latter builds on the “permulation” strategy for trait randomization (Saputra *et al.* 2021) and its implementation relies on the *simpervec()* function from the RERconverge package (Kowalczyk *et al.* 2019). Finally, the *bootstrap* tool determines the iterations returning a CAAS for each position in a MSA to establish the corresponding empirical *P*-value for the detection of a CAAS in that position. Both the discovery and the bootstrap tools are designed to be launched on single MSAs, in order to allow the user to parallelize the workflow for large protein sets.

## 3. Usage and testing

CAAStools users should take special care when designing the analysis and interpreting the results. The comparison should be made between species with diverging values of a convergent phenotype. Each DG should include species with comparable phenotype values from different lineages. The values between the two DGs must diverge, ideally representing the extreme top and bottom values in a continuous distribution or different binary conditions. The resulting output will consist of a list of positions where at least one DG shares the same amino acid, which differs from those found in the other DG. Depending on the DGs selected (often limited by the available phenotypic and genetic information), this outcome may be influenced by various uninformative sources of sequence variability, such as convergent noise and identity-by-descent. Therefore, it is advisable to complement the CAAS analysis with other approaches that have different limitations, such as ancestral state reconstruction (Royer-Carenzi and Didier, 2016), selection studies (Kosakovsky Pond *et al.*, 2020), or dN/dS analysis (Yang, 1997). For e.g., we tested CAAStools on the dataset from Farré *et al.*, (2021). The details of this test are reported in Supplementary 3. The full dataset is available in the /test folder within the CAAStools repository.

## Supplementary Material

btad623_Supplementary_Data
