# Supplementary material for: CAAStools: a toolbox to identify and test Convergent Amino Acid Substitutions
Source: Bioinformatics. 2023 Oct 16;39(10):btad623. doi: 10.1093/bioinformatics/btad623 (PMC10598582; doi:10.1093/bioinformatics/btad623)
Supplement: btad623_Supplementary_Data [file btad623_supplementary_data.zip › Minor.supplementary.ctmanuscript.docx]

**CAAStools, a toolbox to identify and test Convergent Amino Acid Substitutions.**

**Supplementary information.**

**Supplementary 1. CAAS Discovery algorithm.**

Given two Discovery Groups (DGs, Foreground and Background groups, FG and BG, respectively), the discovery tool recognizes as CAAS all those substitutions that meet two requirements. Let *A* be an MSA of *q* sequences of length *t.* We can describe A as an array of *t* positions [1]. Each position (*pos_i_*) will consist of a set of N different amino acids, *a*, with absolute frequency (or count), *f*, where *NS* is the total number of symbols in the alignment [1].

[1] $A=\left( {pos}_{1} {pos}_{2} \ldots{pos}_{t} \right)$ ; ${pos}_{i}= a_{1} f_{1} a_{2} f_{2} {\ldots a}_{NS} {\ldots f}_{NS} ; \sum_{1}^{NS} f=q$

The FG and the BG are formalized as sets of different species s_FG_ and s_BG_, with no intersection and size l_FG_ and l_BG_ [2].

[2] $s_{FG}=\left( s_{1} s_{2} \ldots s_{l_{FG}} \right)$ ; $s_{BG}=\left( s_{1} s_{2} \ldots s_{l_{BG}} \right)$

$s_{FG}\cap s_{BG}=\emptyset$

In each alignment position, $s_{FG}$ and $s_{BG}$ are associated with two sets of amino acids, ${fg(pos}_{i})$ and ${bg(pos}_{i})$, with length $w_{FG}$ and $w_{BG}$.

[3] ${fg(pos}_{i})= a_{1} f_{1} a_{2} f_{2} {\ldots a}_{w_{FG}} {\ldots f}_{w_{FG}}$ ; ${bg(pos}_{i})= a_{1} f_{1} a_{2} f_{2} {\ldots a}_{w_{BG}} {\ldots f}_{w_{BG}}$

CAAStools identifies a CAAS when three conditions are met [4]. First, the two groups must share no amino acids. This means that all the species in the FG need to have different AAs than the species in the BG. Second, at least one of the two DGs must share (or “converge to”) the same amino acid. Also, the CAAS is detected if at least one amino acid is associated to both DGs

[4] ${CAAS}_{i}\{{fg(pos}_{i}) \cap{bg(pos}_{i})=\emptyset w_{FG}== 1 or w_{BG} == 1 w_{FG}> 0 and w_{BG} > 0$

The combination of these three rules defines 3 different mutation *patterns*. We define *pattern 1* when the DGs converge to two different amino acids ($w_{FG}= 1; w_{BG} = 1$). The *pattern 2* will be verified as the FG converges to one amino acid, but the BG will be associated with different amino acids ($w_{FG}= 1; w_{BG} > 1$). *Pattern 3* will consist in the opposite situation, or else when the FG is associated with different amino acids, whilst the BG converges to a single amino acid ($w_{FG}> 1; w_{BG} = 1$). **Supplementary Table 1** summarizes the different mutation patterns and the meeting of requirements for CAAS identification.

***Supplementary Table 1.*** *Mutation patterns and associated program decisions on CAAS assignment.*

| **Discovery Groups** | | **Difference between DGs** | **Convergence in** | | **Pattern** |
| --- | --- | --- | --- | --- | --- |
| **FG** | **BG** |  | **FG** | **BG** |  |
| KV | K | NO | NO | YES | Not a CAAS (No difference) |
| M | TM | NO | YES | NO | Not a CAAS (No difference) |
| MK | VE | YES | NO | NO | Not a CAAS (No convergence) |
| K | V | YES | YES | YES | **Pattern 1** (Both convergent) |
| K | VM | YES | YES | NO | **Pattern 2** (FG convergent, BG multiple) |
| KE | W | YES | NO | YES | **Pattern 3** (FG multiple, BG convergent) |

**Supplementary 2. CAAS discovery statistical testing**

CAAStools calculates an empirical p-value for each CAAS prediction. This p-value is equal to the probability of obtaining a CAAS with random species, and under the same conditions as the CAAS discovery (size of the DGs, maximum permitted gaps and missing species). Following the MSA description in [1], we’ll consider a couple of DG (*FG* and *BG*) of size $l_{FG}$ and $l_{BG}$, as formalized in [2]. The probability to obtain a CAAS from random species is calculated as the probability of extracting concomitantly $k_{FG}$ and$k_{BG}$ objects from a population of size N over a number of extractions *n*, provided the conditions in [4], i.e. $k_{FG} \cap k_{BG} = \emptyset$ and ${wk}_{FG}== 1 or {wk}_{BG} == 1$ where *wk* is the number of symbols in the resampling *k*. This probability can be calculated through the probability mass function from the hypergeometric distribution [5].

[5] $P\left( k \right)= \frac{\left( \frac{K}{k} \right)\left( \frac{N-k}{n-k} \right)}{\left( \frac{N}{n} \right)}=Hyp(N,K,k,n)$

$P\left( CAAS \right)= P(FG) * P(BG)$

$\{P\left( FG \right)= Hyp\left( N_{FG},K_{FG},k_{FG},n_{FG} \right) N_{FG}= q ; k_{FG}=l_{FG} - {null}_{FG} n_{FG} = k_{FG}$

$\{P\left( BG \right)= Hyp\left( N_{BG},K_{BG},k_{BG},n_{NG} \right) N_{BG}= q - l_{FG} k_{BG}=l_{BG} - {null}_{BG} n_{BG} = k_{BG}$

Note that the size of the population N in *P(FG)* differs from the one considered in *P(BG)*. In the first case, the probability of obtaining a convergence in the *FG* is calculated on the total number of sequences in the alignment. In *BG*, the size considered is the difference between the total number of sequences in the alignment *q* and the size of the other group ($q - l_{BG}$), ($q - l_{FG}$), as the two events are concomitant but not independent. The number of extractions $k_{FG}$ and $k_{BG}$ are equal to the number of the difference between the size of the DGs and the number of indels and missing species allowed by the user (*null*). The terms $K_{FG}$ and $K_{BG}$ represent the number of successes in the population. In [6], [7] and [8], we see how this value can be calculated considering all the possible combinations of amino acid symbols that meet the requirements for CAAS detection [4].

[6] ${C_{P1,2} = \{K}_{FG}=\left[ f_{j} \right]; K_{BG}=\left[ q-f_{j} \right] \forall a_{j} \in{pos}_{i}\}$

$$C_{P1,2} = [(K_{{FG}_{1}}; K_{{BG}_{1}}),(K_{{FG}_{2}}; K_{{BG}_{2}})\ldots(K_{{FG}_{z}}; K_{{BG}_{z}})]$$

[7] ${C_{P1,3} = \{K}_{FG}=\left[ {q - f}_{j} \right]; K_{BG}=\left[ f_{j} \right] \forall a_{j} \in{pos}_{i}\}$

$$C_{P1,3} = [(K_{{FG}_{1}}; K_{{BG}_{1}}),(K_{{FG}_{2}}; K_{{BG}_{2}})\ldots(K_{{FG}_{z}}; K_{{BG}_{z}})]$$

[8] ${C_{P1} = \{K}_{FG}=\left[ f_{j} \right]; K_{BG}=\left[ f_{h} \right] \forall a_{j},a_{k} \in{pos}_{i}\}$

$$C_{P1} = [(K_{{FG}_{1}}; K_{{BG}_{1}}),(K_{{FG}_{2}}; K_{{BG}_{2}})\ldots(K_{{FG}_{z}}; K_{{BG}_{z}})]$$

These combinations are based on patterns (*P*). Note that $C_{P1,2}$ and $C_{P1,3}$ overlap, and that the intersection coincides with $C_{P1}$. We can now calculate the CAAS probability separately for each pattern [9].

[9] $P({CAAS}_{P1,3}) = \sum_{x=1}^{z} Hyp(N_{FG},K_{{FG}_{x}},k_{FG},n_{FG}) * Hyp(N_{BG},K_{{BG}_{x}},k_{BG},n_{NG})$

$P({CAAS}_{P1,2}) = \sum_{x=1}^{z} Hyp(N_{FG},K_{{FG}_{x}},k_{FG},n_{FG}) * Hyp(N_{BG},K_{{BG}_{x}},k_{BG},n_{NG})$

$P({CAAS}_{P1}) = \sum_{x=1}^{z} Hyp(N_{FG},K_{{FG}_{x}},k_{FG},n_{FG}) * Hyp(N_{BG},K_{{BG}_{x}},k_{BG},n_{NG})$

The probability to obtain a CAAS in position ${pos}_{i}$ is hence calculated as it follows:

[10] ${pvalue}_{{pos}_{i}}= P({CAAS}_{{pos}_{i}}) = P({CAAS}_{P1,3}) + P({CAAS}_{P1,2}) - P({CAAS}_{P1})$

**2.1 Correction for discovery groups of equal size.**

If the species found in the alignment are the same for FG and BG sizes ($l_{FG}=l_{BG}$), the probability of retrieving pattern 2 and pattern 3 are equal. In this case, the p-value is equal to the $P({CAAS}_{P1,2})$.

[11] $\{{pvalue}_{{pos}_{i}}= P({CAAS}_{{pos}_{i}}) = P\left( {CAAS}_{P1,2} \right) if l_{FG}=l_{BG}$

**Supplementary 3. CAAS discovery from Farré et al., 2021.**

As a test run for CAAStools, we repeated the CAAS discovery from the results published by Farré et al., in 2021 and entitled “*Comparative Analysis of Mammal Genomes Unveils Key Genomic Variability for Human Life Span*” (DOI: [10.1093/molbev/msab219](https://doi.org/10.1093/molbev/msab219)). In this work, 13,035 MSA from UCSC public database (<https://genome.ucsc.edu/>, accessed August, 2019) were scanned to find CAAS between two groups of species with divergent maximum lifespan. The “long lived” group is formed by *Homo sapiens* (hg38), *Nomascus leucogenys* (nomLeu3), *Heterocephalus glaber* (hetGla2), *Myotis davidii* (myoDav1), *Myotis lucifugus* (myoLuc2), *Eptesicus fuscus* (eptFus1). The “short lived” group is formed by *Mesocricetus auratus* (mesAur1), *Rattus norvegicus* (rn6), *Pantholops hodgsonii* (panHod1), *Sorex araneus* (sorAra2), *Condylura cristata* (conCri1), *Monodelphis domestica* (monDom5). Farré et al., filtered the results from CAAS discovery to those CAAS having no gaps or missing species, and focused their analysis on the CAAS of scenarios 1 and 2, which correspond to patter 1 and 2 in CAAStools terminology.

We have repeated this analysis under the same conditions, filtering for pattern 1 and 2 and for no gaps in foreground (*short-lived* group) and background (*long-lived* group). The results (*Supplementary dataset 1***)** and the phenotype configuration (*Supplementary dataset 2*) are available in the supplementary.material.xls spreadsheet. Our analysis confirmed the identification of 2737 mutations in 2004 MSA.

**Supplementary 4. An example of p-value calculation and correction via bootstrap from *Farré et al. 2021* dataset.**

The gene BRCA2 (RefSeq code: *NM_000059*) is part of the results published by Farré et al. in 2021. In that analysis, authors selected only those positions that were associated with no gaps in both Foreground and Background, obtaining 7 CAAS from this gene. Here, we repeated the CAAS detection without any filtering for gaps or missing species on the BRCA2 gene. Then, we used the simulation tool to generate 1,000 simulated traits for each simulation mode (random, random with phylogeny restriction and Brownian motion). We finally ran a bootstrap for each strategy and compared the resulting p-values with the one calculated by the discovery tool. The result is shown in *Supplementary Table 2*, whilst the extended CAAStools discovery output can be found in *Supplementary Dataset 3*.

***Supplementary Table 2.*** *P-value comparison on CAAS found on BRCA2.*

| **Gene** | **Position in MSA** | **Substitution** | **p-value** | | | |
| --- | --- | --- | --- | --- | --- | --- |
|  |  |  | **Hypergeometric** | **Random** | **Phylogeny-restricted** | **Brownian motion** |
| NM_000059 | 46 | A/PSV | 0.00111290839 | 0.002 | 0.086 | 0.016 |
| NM_000059 | 258 | R/GKQT | 0.0001146674319 | 0 | 0.033 | 0.022 |
| NM_000059 | 481 | L/IMPT | 0.0002223201488 | 0 | 0.016 | 0.006 |
| NM_000059 | 483 | V/GILT | 0.0002482399421 | 0.001 | 0.154 | 0.01 |
| NM_000059 | 631 | AIL/T | 0.0003309226089 | 0.001 | 0.013 | 0.024 |
| NM_000059 | 953 | E/DK | 0.02510771161 | 0.003 | 0.014 | 0.055 |
| NM_000059 | 979 | D/EGN | 0.0037519556 | 0 | 0.421 | 0.071 |
| NM_000059 | 1172 | I/ALPTV | 0.007452886308 | 0.001 | 0.144 | 0.026 |
| NM_000059 | 1216 | R/GKS | 0.0001084014692 | 0 | 0.059 | 0.009 |
| NM_000059 | 1297 | I/AFKNTV | 0.0002223401233 | 0 | 0.011 | 0.002 |
| NM_000059 | 1361 | H/CGQRY | 0.000565102993 | 0.002 | 0.076 | 0.004 |
| NM_000059 | 1548 | K/ET | 0.002094076584 | 0.006 | 0.355 | 0.061 |
| NM_000059 | 1585 | T/N | 0.1699568024 | 0.038 | 0.383 | 0.159 |
| NM_000059 | 1858 | I/V | 0.01704761083 | 0.047 | 0.226 | 0.106 |
| NM_000059 | 1935 | M/IKV | 0.0002880415021 | 0.002 | 0.058 | 0.004 |
| NM_000059 | 2012 | K/EMRT | 0.0009116846684 | 0.001 | 0.013 | 0.01 |
| NM_000059 | 2039 | I/L | 0.1171992697 | 0.089 | 0.254 | 0.167 |
| NM_000059 | 2261 | M/ART | 0.006530180592 | 0.007 | 0.082 | 0.069 |
| NM_000059 | 3418 | Z/QS | 0.006622240227 | 0.001 | 0.037 | 0.039 |

The random resampling returns p-values that compare to those calculated by the hypergeometric function from the discovery tool (*hypergeometric*). Besides, the hypergeometric p-value reflects the probability to find a CAAS in a certain position with random species. The difference between hypergeometric and random bootstrap relies on the sets of species that are considered for resampling. Whilst the hypergeometric function p-value is calculated on the species that are present in the alignment, the random sampling is based on the species that are present in the phylogenetic tree. The user might be motivated to choose the random resampling if the number of species in the alignment differs remarkably from the number of species in the phylogeny. Note that in our example, the number of species in the alignment equals the number of species in the phylogeny (Farré et al., 2021).

As we apply phylogenetic constraints to random re-samplings, we observe a radical increase of the p-values. This strategy, indicated as “*phylogeny-restricted*”, is still based on the random selection of species. Differently from the “*random*” resampling, however, the *phylogeny-restricted* strategy limits the species extraction to some specific clades. These clades correspond to the ones that are present in the DGs used in the discovery tool that serve as “template”. This limitation corresponds to a radical reduction of the probability space and to an increase of the p-values (Supplementary Table 2). In this case, the p-values reflect the probability to find aleatory convergences in the clades used for CAAS discovery.

Finally, the resampling tool allows to simulate DGs through a Brownian-motion stochastic process (Supplementary Table 2). In this case, the program will simulate a neutral phenotype distribution over the phylogeny, to form the DGs by selecting species with top and bottom values. With this approach, phylogenetically closer species tend to exhibit similar phenotype values (Saputra et al., 2021). The simulated traits will hence compare close species from different partitions of the phylogeny. This represents an obvious reduction of the probability space, as not all the species combinations are equiprobable. Also, it tends to compare species that come from different lineages and that are more prone to share different amino-acids. The p-values are hence higher than those calculated by both the discovery tool (using the hypergeometric method) and the p-values simulated in the 'random' strategy. Conversely, the p-values simulated through the “phylogeny-restricted” strategy – which reduces dramatically the probability space- are tendentially higher.

Further details on the statistical testing are provided in CAAStools documentation (<https://github.com/linudz/caastools/blob/main/README.md>).
